# Healthcare Resource Utilization After Surgical Treatment of Cancer: Value of Minimally Invasive Surgery

**DOI:** 10.1007/s00464-022-09189-8

**Published:** 2022-04-21

**Authors:** Rocco Ricciardi, Robert Neil Goldstone, Todd Francone, Matthew Wszolek, Hugh Auchincloss, Alexander de Groot, I.-Fan Shih, Yanli Li

**Affiliations:** 1 Section of Colon & Rectal Surgery, Massachusetts General Hospital, Harvard Medical School, 15 Parkman Street, WACC 460, Boston, MA USA; 2 Department of Urology, Massachusetts General Hospital, Harvard Medical School, Boston, MA USA; 3 Division of Thoracic Surgery, Massachusetts General Hospital, Harvard Medical School, Boston, MA USA; 4 Global Access, Value, & Economics, Intuitive Surgical, Sunnyvale, CA USA

**Keywords:** Length of stay, Readmission, Cancer, Minimally invasive surgery, Robotic-assisted surgery, Laparoscopic surgery

## Abstract

**Background:**

As the US healthcare system moves towards value-based care, hospitals have increased efforts to improve quality and reduce unnecessary resource use. Surgery is one of the most resource-intensive areas of healthcare and we aim to compare health resource utilization between open and minimally invasive cancer procedures.

**Methods:**

We retrospectively analyzed cancer patients who underwent colon resection, rectal resection, lobectomy, or radical nephrectomy within the Premier hospital database between 2014 and 2019. Study outcomes included length of stay (LOS), discharge status, reoperation, and 30-day readmission. The open surgical approach was compared to minimally invasive approach (MIS), with subgroup analysis of laparoscopic/video-assisted thoracoscopic surgery (LAP/VATS) and robotic (RS) approaches, using inverse probability of treatment weighting.

**Results:**

MIS patients had shorter LOS compared to open approach: − 1.87 days for lobectomy, − 1.34 days for colon resection, − 0.47 days for rectal resection, and − 1.21 days for radical nephrectomy (all *p* < .001). All MIS procedures except for rectal resection are associated with higher discharge to home rates and lower reoperation and readmission rates. Within MIS, robotic approach was further associated with shorter LOS than LAP/VATS: − 0.13 days for lobectomy, − 0.28 days for colon resection, − 0.67 days for rectal resection, and − 0.33 days for radical nephrectomy (all *p* < .05) and with equivalent readmission rates.

**Conclusion:**

Our data demonstrate a significant shorter LOS, higher discharge to home rate, and lower rates of reoperation and readmission for MIS as compared to open procedures in patients with lung, kidney, and colorectal cancer. Patients who underwent robotic procedures had further reductions in LOS compare to laparoscopic/video-assisted thoracoscopic approach, while the reductions in LOS did not lead to increased rates of readmission.

**Supplementary Information:**

The online version contains supplementary material available at 10.1007/s00464-022-09189-8.

Cancer is a leading cause of death worldwide with nearly 20 million new cases and 10 million cancer-related deaths globally in 2020 [1]. While cancer treatment varies depending on the location, stage, and type of cancer, surgical resection is a crucial part of multimodality treatment for many solid tumors. Over the past few decades, there has been a shift of surgical treatment to more minimally invasive approaches due to the smaller incision, less pain, and a quicker recovery [2–5]. Innovation in minimally invasive surgery (MIS) has led to the development of precision robotic systems aimed at improving surgical conduct. Smoother instrument dexterity improved three-dimensional vision, instrument articulation, and enhanced accessibility to difficult spaces are significant enhancements of the robotic system when compared to other MIS options. These enhancements have led to the adoption and diffusion of robotic-assisted surgery to a wide range of specialty fields within surgery.

As surgery is one of the most resource-intensive areas of clinical medicine, there is a growing trend for quality improvement initiatives to improve the efficiency, quality, and safety of surgical care, to reduce unnecessary consumption of resources, and to increase patient satisfaction [6]. Some commonly used indicators to measure surgical care resource utilization include hospital length of stay (LOS), reoperation, and readmission. With the increasing adoption of MIS, especially robotic surgery, it is necessary to better understand their impact on healthcare resource utilization and quality of surgical care. This is of greater importance in the context of the COVID-19 pandemic given the need to free up hospital beds, staff shortages, and other competing resource needs.

Some prior studies have evaluated healthcare resource utilization after MIS; however, they tended to focus on specific procedures or were performed in single institutions [7, 8]. This limits their generalizability to the broad range of surgical oncological procedures performed at the national level. The aim of this study was to leverage a national hospital discharge database in the US.

## Methods

### Data source

The Premier Healthcare Database (PHD) was used for this study. The database contains service-level information for hospital-based inpatient admissions and outpatient encounters for over 231 million patients in the United States. Clinical, billing, and financial information can be tracked for patients within the same hospital in the database [9]. Institutional Review Board approval was not necessary for this study because PHD is commercially available and de-identified.

### Study population

Hospital encounters for adults 18 years of age and older were included in the study if the patient underwent one of the following primary, elective inpatient procedures between January 1, 2014 and December 31, 2019 using either an open, laparoscopic/video-assisted thoracoscopic surgery (LAP/VATS), or robotic-assisted (RAS) surgical approach: (1) colon resection for colon cancer, (2) rectal resection for rectal cancer, (3) lobectomy for primary lung cancer, or (4) radical nephrectomy for kidney cancer. Procedures and their corresponding surgical approaches were defined using International Classification of Diseases, Ninth Revision (ICD-9) codes; ICD-10 Codes; Current Procedural Terminology (CPT) codes; and hospital billing records (Supplemental Table 1). An encounter was excluded from the analysis if the corresponding procedure’s operating room time or total cost was less than or equal to zero minutes or dollars, respectively.

### Study outcomes

The primary outcome for this analysis was length of stay (LOS), which is directly captured in the PHD and is calculated as the discharge date minus the admission date. Secondary outcomes included reoperation during hospital stay, discharge to home, and 30-day readmission rates. Reoperation was defined as any return to operating room billing record after index surgery.

### Study covariates

Patient, surgeon, and hospital characteristics were used as covariates in the analysis. Patient characteristics included age, gender, race/ethnicity, insurance, Charlson Comorbidity Index (CCI; excluding cancer), presence of metastasis, obesity, smoking history, and year of surgery. Surgeon characteristics included surgeon specialty and surgeon procedure volume. Hospital covariates included hospital procedure volume, geographic region, teaching status, rural/urban, and hospital bed size.

### Statistical analysis

All descriptive and statistical testing analyses were conducted by procedure comparing open surgical approach to MIS, and LAP/VATS to RAS. Unstratified descriptive statistics were also calculated across all procedures. For both the crude and adjusted analyses, the gtsummary v1.4.2 package in R was used to calculate frequencies and proportions for categorical outcomes and covariates, and means, medians, standard deviations, and interquartile ranges for continuous outcomes and covariates.

Adjusted analyses were achieved using Inverse Probability of Treatment Weighting (IPTW) through the WeightIt v0.12.0 package in R. Stabilized propensity score weights were used to estimate the average treatment effect and all patient, surgeon, and hospital covariates were used to create balance between the groups [10]. A covariate was considered balanced if the absolute value of the standardized mean difference after adjustment was less than 0.10. Using the IPTW-adjusted data, adjusted mean differences and odds ratios were calculated. A gamma regression with an identity link was used to calculate the mean difference and 95% confidence interval between comparison groups for LOS. A logistic regression model was used to calculate the odds ratio and 95% confidence interval between comparison groups for reoperation, discharge to home, and 30-day readmission rates. Mean differences and odds ratios were considered significant if the p-values were less than 0.05. For the lobectomy procedure comparing open surgical approach to MIS, surgeon procedure volume and hospital procedure volume were added as additional adjustment variables to the models because the absolute values of the standardized mean differences for both covariates after IPTW were not less than 0.10. In the sensitivity analysis, we assessed the conversion to open surgery, ICU admission for at least 1 day, ICU admission for at least two days, and mechanical ventilation usage. All analyses were conducted using R version 4.1.1.

## Results

From 2014 to 2019, a total of 122,815 patients who underwent surgical oncological procedures were extracted from PHD: 33,383 (27.2%) lobectomy, 51,948 (42.3%) colon resection, 11,052 (9.0%) rectal resection, and 26,432 (21.5%) radical nephrectomy. While the adoption of minimally invasive surgery (LAP/VATS and RAS) is similar across procedures (between 62.6% and 66.3%), the adoption of RAS within MIS varies: 53.0% for rectal resection, 46.5% for radical nephrectomy, 37.7% for lobectomy and 24.9% for colon resection. Baseline characteristics prior to IPTW are shown in Table 1 and 2. After IPTW, patient, surgeon, and hospital characteristics were comparable (with standardized mean difference < 0.1; Supplementary Table 1 and 2), except for surgeon and hospital procedure volumes in open vs MIS lobectomies.

**Table 1:** Demographic and preoperative characteristics, open vs. Minimally invasive surgical approach (MIS): Before inverse probability treatment weighting (IPTW)

Characteristic	Lobectomy			Colon resection			Rectal resection			Radical nephrectomy		
	Open, *N* = 12,110	MIS, *N* = 21,273	Std Diff	Open, *N* = 17,495	MIS, *N* = 34,453	Std Diff	Open, *N* = 4118	MIS, *N* = 6934	Std Diff	Open, N = 9873	MIS, N = 16,559	Std Diff
Age groups, *n* (%)												
18–44 years	132 (1.1)	189 (0.9)	0.020	682 (3.9)	1416 (4.1)	0.011	282 (6.8)	496 (7.2)	0.012	632 (6.4)	1102 (6.7)	0.010
45–54 years	907 (7.5)	1287 (6.0)	0.057	1929 (11.0)	4633 (13.4)	0.074	759 (18.4)	1487 (21.4)	0.076	1516 (15.4)	2565 (15.5)	0.004
55–64 years	3340 (27.6)	5383 (25.3)	0.052	3646 (20.8)	7557 (21.9)	0.027	1187 (28.8)	2031 (29.3)	0.010	2790 (28.3)	4425 (26.7)	0.034
65 +	7731 (63.8)	14,414 (67.8)	0.083	11,238 (64.2)	20,847 (60.5)	0.077	1890 (45.9)	2920 (42.1)	0.076	4935 (50.0)	8467 (51.1)	0.023
Gender, Male, *n* (%)	5909 (48.8)	9389 (44.1)	0.094	8376 (47.9)	17,024 (49.4)	0.031	2497 (60.6)	4256 (61.4)	0.015	6280 (63.6)	10,355 (62.5)	0.022
Race/ethnicity, *n* (%)												
White	10,255 (84.7)	17,685 (83.1)	0.042	13,867 (79.3)	26,961 (78.3)	0.025	3297 (80.1)	5548 (80.0)	0.001	7,596 (76.9)	12,631 (76.3)	0.016
African American	865 (7.1)	1617 (7.6)	0.018	1834 (10.5)	3483 (10.1)	0.012	290 (7.0)	504 (7.3)	0.009	929 (9.4)	1587 (9.6)	0.006
Hispanic	303 (2.5)	913 (4.3)	0.099	729 (4.2)	1837 (5.3)	0.055	272 (6.6)	402 (5.8)	0.034	659 (6.7)	1138 (6.9)	0.008
Other	687 (5.7)	1058 (5.0)	0.031	1065 (6.1)	2172 (6.3)	0.009	259 (6.3)	480 (6.9)	0.026	689 (7.0)	1203 (7.3)	0.011
Insurance type, *n* (%)												
Medicare	7965 (65.8)	14,410 (67.7)	0.042	10,998 (62.9)	20,215 (58.7)	0.086	1875 (45.5)	2874 (41.4)	0.082	5129 (51.9)	8832 (53.3)	0.028
Medicaid	870 (7.2)	1231 (5.8)	0.057	1057 (6.0)	1639 (4.8)	0.057	439 (10.7)	690 (10.0)	0.023	725 (7.3)	1147 (6.9)	0.016
Commercial	2802 (23.1)	4966 (23.3)	0.005	4764 (27.2)	11,410 (33.1)	0.129	1584 (38.5)	3030 (43.7)	0.107	3499 (35.4)	5860 (35.4)	0.001
Other	473 (3.9)	666 (3.1)	0.042	676 (3.9)	1189 (3.5)	0.022	220 (5.3)	340 (4.9)	0.020	520 (5.3)	720 (4.3)	0.043
Charlson Comorbidity Index (CCI), *n* (%)												
CCI = 0	3559 (29.4)	7657 (36.0)	0.141	8244 (47.1)	19,534 (56.7)	0.193	2356 (57.2)	4315 (62.2)	0.102	5239 (53.1)	9468 (57.2)	0.083
CCI = 1	4861 (40.1)	8201 (38.6)	0.033	2311 (13.2)	4696 (13.6)	0.012	536 (13.0)	803 (11.6)	0.044	1854 (18.8)	3220 (19.4)	0.017
CCI ≥ 2	3690 (30.5)	5415 (25.5)	0.112	6940 (39.7)	10,223 (29.7)	0.211	1226 (29.8)	1816 (26.2)	0.080	2780 (28.2)	3871 (23.4)	0.110
Metastasis, n (%)	1898 (15.7)	2478 (11.6)	0.117	4468 (25.5)	5725 (16.6)	0.220	848 (20.6)	1194 (17.2)	0.086	1345 (13.6)	1081 (6.5)	0.237
Obese or overweight, n (%)	1753 (14.5)	2878 (13.5)	0.027	3369 (19.3)	6685 (19.4)	0.004	679 (16.5)	1242 (17.9)	0.038	2181 (22.1)	3726 (22.5)	0.010
Current or former smoker, n (%)	9625 (79.5)	16,219 (76.2)	0.078	6506 (37.2)	12,326 (35.8)	0.029	1603 (38.9)	2735 (39.4)	0.011	3952 (40.0)	6702 (40.5)	0.009
Surgeon specialty, *n* (%)												
Procedure specialist	10,140 (83.7)	18,245 (85.8)	0.057	3602 (20.6)	11,125 (32.3)	0.268	1810 (44.0)	3512 (50.6)	0.134	9056 (91.7)	15,613 (94.3)	0.101
General surgery	832 (6.9)	1736 (8.2)	0.049	11,785 (67.4)	19,900 (57.8)	0.199	1924 (46.7)	2795 (40.3)	0.130	125 (1.3)	88 (0.5)	0.078
Other/Unknown	1138 (9.4)	1292 (6.1)	0.125	2108 (12.0)	3428 (9.9)	0.067	384 (9.3)	627 (9.0)	0.010	692 (7.0)	858 (5.2)	0.076
Surgeon volume, *n* (%)												
Low	5434 (44.9)	5314 (25.0)	0.427	5319 (30.4)	9511 (27.6)	0.062	941 (22.9)	1681 (24.2)	0.033	4218 (42.7)	5155 (31.1)	0.242
Medium	4790 (39.6)	6270 (29.5)	0.213	6772 (38.7)	10,849 (31.5)	0.152	1411 (34.3)	1917 (27.6)	0.144	4031 (40.8)	6277 (37.9)	0.060
High	1886 (15.6)	9689 (45.5)	0.688	5404 (30.9)	14,093 (40.9)	0.210	1766 (42.9)	3336 (48.1)	0.105	1624 (16.4)	5127 (31.0)	0.346
Hospital volume, *n* (%)												
Low	5426 (44.8)	5729 (26.9)	0.379	7469 (42.7)	11,076 (32.1)	0.219	1069 (26.0)	1535 (22.1)	0.090	4374 (44.3)	6410 (38.7)	0.114
Medium	4728 (39.0)	6775 (31.8)	0.151	5460 (31.2)	12,549 (36.4)	0.110	1401 (34.0)	2565 (37.0)	0.062	3237 (32.8)	5549 (33.5)	0.015
High	1956 (16.2)	8769 (41.2)	0.577	4566 (26.1)	10,828 (31.4)	0.118	1648 (40.0)	2834 (40.9)	0.017	2262 (22.9)	4600 (27.8)	0.112
Teaching hospital, *n* (%)	6138 (50.7)	13,417 (63.1)	0.252	7857 (44.9)	16,231 (47.1)	0.044	2199 (53.4)	3765 (54.3)	0.018	4994 (50.6)	8645 (52.2)	0.033
Urban Region, *n* (%)	11,208 (92.6)	19,548 (91.9)	0.025	14,811 (84.7)	30,662 (89.0)	0.129	3690 (89.6)	6339 (91.4)	0.062	8987 (91.0)	15,110 (91.2)	0.008
Geographic region, *n* (%)												
Midwest	3148 (26.0)	4486 (21.1)	0.116	4406 (25.2)	7497 (21.8)	0.081	985 (23.9)	1585 (22.9)	0.025	2115 (21.4)	3791 (22.9)	0.035
Northeast	1032 (8.5)	4503 (21.2)	0.361	2560 (14.6)	5715 (16.6)	0.054	558 (13.6)	1020 (14.7)	0.033	1243 (12.6)	2654 (16.0)	0.098
South	6165 (50.9)	9317 (43.8)	0.143	8050 (46.0)	16,056 (46.6)	0.012	1860 (45.2)	3201 (46.2)	0.020	4925 (49.9)	7471 (45.1)	0.096
West	1765 (14.6)	2967 (13.9)	0.018	2479 (14.2)	5185 (15.0)	0.025	715 (17.4)	1128 (16.3)	0.029	1590 (16.1)	2643 (16.0)	0.004
Hospital bed size, *n* (%)												
0–299 beds	2537 (20.9)	3534 (16.6)	0.111	6242 (35.7)	11,104 (32.2)	0.073	1066 (25.9)	1723 (24.8)	0.024	2572 (26.1)	4737 (28.6)	0.057
300–499 beds	4548 (37.6)	6133 (28.8)	0.186	5472 (31.3)	10,118 (29.4)	0.042	1289 (31.3)	2148 (31.0)	0.007	3187 (32.3)	4796 (29.0)	0.072
500 + beds	5025 (41.5)	11,606 (54.6)	0.264	5781 (33.0)	13,231 (38.4)	0.112	1763 (42.8)	3063 (44.2)	0.028	4114 (41.7)	7026 (42.4)	0.015
Year of surgery, *n* (%)												
2014	2421 (20.0)	3039 (14.3)	0.152	3292 (18.8)	6179 (17.9)	0.023	609 (14.8)	1010 (14.6)	0.006	2304 (23.3)	2233 (13.5)	0.256
2015	2518 (20.8)	3218 (15.1)	0.148	3135 (17.9)	6060 (17.6)	0.009	776 (18.8)	1070 (15.4)	0.091	2137 (21.6)	2590 (15.6)	0.155
2016	2230 (18.4)	3363 (15.8)	0.069	3298 (18.9)	5399 (15.7)	0.084	934 (22.7)	1270 (18.3)	0.108	1458 (14.8)	2916 (17.6)	0.077
2017	1945 (16.1)	3767 (17.7)	0.044	2881 (16.5)	5942 (17.2)	0.021	734 (17.8)	1278 (18.4)	0.016	1463 (14.8)	3048 (18.4)	0.097
2018	1725 (14.2)	3931 (18.5)	0.115	2622 (15.0)	5550 (16.1)	0.031	561 (13.6)	1243 (17.9)	0.118	1289 (13.1)	2884 (17.4)	0.122
2019	1271 (10.5)	3955 (18.6)	0.231	2267 (13.0)	5323 (15.5)	0.071	504 (12.2)	1063 (15.3)	0.090	1222 (12.4)	2888 (17.4)	0.143
Colon resection type, *n* (%)												
Left colectomy	NA	NA		5876 (33.6)	11,865 (34.4)	0.018	NA	NA		NA	NA	
Right colectomy	NA	NA		11,619 (66.4)	22,588 (65.6)	0.018	NA	NA		NA	NA	

*Std diff* standardized mean differences

**Table 2:** Demographic and preoperative characteristics, Laparoscopic/Video-assisted thoracoscopic surgery (LAP/VATS) vs. Robotic (RAS): Before inverse probability treatment weighting (IPTW)

Characteristic	Lobectomy			Colon resection			Rectal resection			Radical nephrectomy		
	VATS, *N* = 13,260	RAS, *N* = 8013	Std Diff	LAP, *N* = 25,864	RAS, *N* = 8589	Std Diff	LAP, *N* = 3256	RAS, *N* = 3678	Std Diff	LAP, *N* = 8857	RAS, *N* = 7702	Std Diff
Age groups, *n* (%)												
18–44 years	119 (0.9)	70 (0.9)	0.003	1034 (4.0)	382 (4.4)	0.022	226 (6.9)	270 (7.3)	0.016	607 (6.9)	495 (6.4)	0.017
45–54 years	815 (6.1)	472 (5.9)	0.011	3378 (13.1)	1255 (14.6)	0.045	675 (20.7)	812 (22.1)	0.033	1352 (15.3)	1213 (15.7)	0.013
55–64 years	3423 (25.8)	1960 (24.5)	0.031	5673 (21.9)	1884 (21.9)	0.000	945 (29.0)	1086 (29.5)	0.011	2402 (27.1)	2023 (26.3)	0.019
65 +	8903 (67.1)	5511 (68.8)	0.035	15,779 (61.0)	5068 (59.0)	0.041	1410 (43.3)	1510 (41.1)	0.046	4496 (50.8)	3971 (51.6)	0.016
Gender, Male, *n* (%)	5839 (44.0)	3550 (44.3)	0.005	12,622 (48.8)	4402 (51.3)	0.049	2000 (61.4)	2256 (61.3)	0.002	5422 (61.2)	4933 (64.0)	0.059
Race/ethnicity, *n* (%)												
White	11,265 (85.0)	6420 (80.1)	0.128	20,249 (78.3)	6712 (78.1)	0.004	2576 (79.1)	2972 (80.8)	0.042	6758 (76.3)	5873 (76.3)	0.001
African American	999 (7.5)	618 (7.7)	0.007	2646 (10.2)	837 (9.7)	0.016	238 (7.3)	266 (7.2)	0.003	906 (10.2)	681 (8.8)	0.047
Hispanic	393 (3.0)	520 (6.5)	0.167	1333 (5.2)	504 (5.9)	0.031	219 (6.7)	183 (5.0)	0.075	628 (7.1)	510 (6.6)	0.019
Other	603 (4.5)	455 (5.7)	0.051	1636 (6.3)	536 (6.2)	0.004	223 (6.8)	257 (7.0)	0.006	565 (6.4)	638 (8.3)	0.073
Insurance type, *n* (%)												
Medicare	8956 (67.5)	5454 (68.1)	0.011	15,297 (59.1)	4918 (57.3)	0.038	1406 (43.2)	1468 (39.9)	0.066	4678 (52.8)	4154 (53.9)	0.022
Medicaid	783 (5.9)	448 (5.6)	0.014	1248 (4.8)	391 (4.6)	0.013	340 (10.4)	350 (9.5)	0.031	608 (6.9)	539 (7.0)	0.005
Commercial	3086 (23.3)	1880 (23.5)	0.005	8410 (32.5)	3000 (34.9)	0.051	1342 (41.2)	1688 (45.9)	0.095	3191 (36.0)	2669 (34.7)	0.029
Other	435 (3.3)	231 (2.9)	0.023	909 (3.5)	280 (3.3)	0.014	168 (5.2)	172 (4.7)	0.022	380 (4.3)	340 (4.4)	0.006
Charlson Comorbidity Index (CCI), *n* (%)												
CCI = 0	4757 (35.9)	2900 (36.2)	0.007	14,534 (56.2)	5000 (58.2)	0.041	1996 (61.3)	2319 (63.1)	0.036	5157 (58.2)	4311 (56.0)	0.046
CCI = 1	5157 (38.9)	3044 (38.0)	0.019	3549 (13.7)	1147 (13.4)	0.011	371 (11.4)	432 (11.7)	0.011	1726 (19.5)	1494 (19.4)	0.002
CCI ≥ 2	3346 (25.2)	2069 (25.8)	0.014	7781 (30.1)	2442 (28.4)	0.036	889 (27.3)	927 (25.2)	0.048	1974 (22.3)	1897 (24.6)	0.055
Metastasis, *n* (%)	1595 (12.0)	883 (11.0)	0.032	4385 (17.0)	1340 (15.6)	0.037	605 (18.6)	589 (16.0)	0.068	529 (6.0)	552 (7.2)	0.048
Obese or overweight, *n* (%)	1660 (12.5)	1218 (15.2)	0.078	4912 (19.0)	1773 (20.6)	0.041	562 (17.3)	680 (18.5)	0.032	1944 (21.9)	1782 (23.1)	0.028
Current or former smoker, *n* (%)	10,066 (75.9)	6153 (76.8)	0.021	9234 (35.7)	3092 (36.0)	0.006	1291 (39.6)	1444 (39.3)	0.008	3452 (39.0)	3250 (42.2)	0.066
Surgeon specialty, *n* (%)												
Procedure specialist	11,173 (84.3)	7072 (88.3)	0.116	7724 (29.9)	3401 (39.6)	0.206	1463 (44.9)	2049 (55.7)	0.217	8267 (93.3)	7346 (95.4)	0.089
General surgery	1080 (8.1)	656 (8.2)	0.002	15,368 (59.4)	4532 (52.8)	0.134	1470 (45.1)	1325 (36.0)	0.187	57 (0.6)	31 (0.4)	0.033
Other/Unknown	1007 (7.6)	285 (3.6)	0.177	2772 (10.7)	656 (7.6)	0.107	323 (9.9)	304 (8.3)	0.058	533 (6.0)	325 (4.2)	0.082
Surgeon volume, *n* (%)												
Low	3590 (27.1)	1724 (21.5)	0.130	7201 (27.8)	2310 (26.9)	0.021	754 (23.2)	927 (25.2)	0.048	3749 (42.3)	1406 (18.3)	0.543
Medium	4099 (30.9)	2171 (27.1)	0.084	8366 (32.3)	2483 (28.9)	0.075	876 (26.9)	1041 (28.3)	0.031	3885 (43.9)	2392 (31.1)	0.267
High	5571 (42.0)	4118 (51.4)	0.189	10,297 (39.8)	3796 (44.2)	0.089	1626 (49.9)	1710 (46.5)	0.069	1223 (13.8)	3904 (50.7)	0.859
Hospital volume, *n* (%)												
Low	3866 (29.2)	1863 (23.2)	0.135	8561 (33.1)	2515 (29.3)	0.083	858 (26.4)	677 (18.4)	0.192	3788 (42.8)	2622 (34.0)	0.180
Medium	4105 (31.0)	2670 (33.3)	0.051	9250 (35.8)	3299 (38.4)	0.055	1170 (35.9)	1395 (37.9)	0.041	3004 (33.9)	2545 (33.0)	0.019
High	5289 (39.9)	3480 (43.4)	0.072	8053 (31.1)	2775 (32.3)	0.025	1228 (37.7)	1606 (43.7)	0.121	2065 (23.3)	2535 (32.9)	0.215
Teaching hospital, n (%)	8502 (64.1)	4915 (61.3)	0.058	12,150 (47.0)	4081 (47.5)	0.011	1653 (50.8)	2112 (57.4)	0.134	4257 (48.1)	4388 (57.0)	0.179
Urban Region, *n* (%)	12,249 (92.4)	7299 (91.1)	0.047	22,758 (88.0)	7904 (92.0)	0.135	2931 (90.0)	3408 (92.7)	0.094	8009 (90.4)	7101 (92.2)	0.063
Geographic region, *n* (%)												
Midwest	2544 (19.2)	1942 (24.2)	0.123	5646 (21.8)	1851 (21.6)	0.007	667 (20.5)	918 (25.0)	0.107	1663 (18.8)	2128 (27.6)	0.211
Northeast	3020 (22.8)	1483 (18.5)	0.106	4373 (16.9)	1342 (15.6)	0.035	467 (14.3)	553 (15.0)	0.020	1366 (15.4)	1288 (16.7)	0.035
South	5599 (42.2)	3718 (46.4)	0.084	11,825 (45.7)	4231 (49.3)	0.071	1440 (44.2)	1,761 (47.9)	0.073	4255 (48.0)	3216 (41.8)	0.127
West	2097 (15.8)	870 (10.9)	0.146	4020 (15.5)	1165 (13.6)	0.056	682 (20.9)	446 (12.1)	0.239	1573 (17.8)	1070 (13.9)	0.106
Hospital bed size, *n* (%)												
0–299 beds	2026 (15.3)	1508 (18.8)	0.094	8575 (33.2)	2529 (29.4)	0.080	870 (26.7)	853 (23.2)	0.082	2563 (28.9)	2174 (28.2)	0.016
300–499 beds	4141 (31.2)	1992 (24.9)	0.142	7531 (29.1)	2587 (30.1)	0.022	1005 (30.9)	1143 (31.1)	0.005	2880 (32.5)	1916 (24.9)	0.170
500 + beds	7093 (53.5)	4513 (56.3)	0.057	9758 (37.7)	3473 (40.4)	0.056	1381 (42.4)	1682 (45.7)	0.067	3414 (38.5)	3612 (46.9)	0.169
Year of surgery, *n* (%)												
2014	2286 (17.2)	753 (9.4)	0.232	5522 (21.4)	657 (7.6)	0.397	713 (21.9)	297 (8.1)	0.395	1266 (14.3)	967 (12.6)	0.051
2015	2405 (18.1)	813 (10.1)	0.231	5149 (19.9)	911 (10.6)	0.261	662 (20.3)	408 (11.1)	0.256	1475 (16.7)	1115 (14.5)	0.060
2016	2318 (17.5)	1045 (13.0)	0.124	4182 (16.2)	1217 (14.2)	0.056	600 (18.4)	670 (18.2)	0.006	1764 (19.9)	1152 (15.0)	0.131
2017	2333 (17.6)	1434 (17.9)	0.008	4239 (16.4)	1703 (19.8)	0.089	518 (15.9)	760 (20.7)	0.123	1615 (18.2)	1433 (18.6)	0.010
2018	2123 (16.0)	1808 (22.6)	0.167	3604 (13.9)	1946 (22.7)	0.227	404 (12.4)	839 (22.8)	0.276	1404 (15.9)	1480 (19.2)	0.089
2019	1795 (13.5)	2160 (27.0)	0.339	3168 (12.2)	2155 (25.1)	0.334	359 (11.0)	704 (19.1)	0.228	1333 (15.1)	1555 (20.2)	0.135
Colon resection type, *n* (%)												
Left colectomy	NA	NA		8410 (32.5)	3455 (40.2)	0.161	NA	NA		NA	NA	
Right colectomy	NA	NA		17454 (67.5)	5134 (59.8)	0.161	NA	NA		NA	NA	

*Std diff* standardized mean differences

In IPTW-adjusted analyses, MIS approach was associated with shorter LOS for all procedures examined compared to open approach: − 1.87 days (95% CI, − 1.99 to − 1.75) for lobectomy, − 1.34 days (95% CI, − 1.43 to − 1.26) for colon resection, − 0.47 days (95% CI, − 0.70 to − 0.24) for rectal resection, and − 1.21 days (95% CI, − 1.30 to − 1.11) for radical nephrectomy (all *p* < 0.001; Table 3). Within MIS, robotic approach was further associated with shorter LOS than LAP/VATS: − 0.13 days (95% CI, − 0.25 to − 0.01) for lobectomy, − 0.28 days (95% CI, − 0.37 to − 0.18) for colon resection, − 0.67 days (95% CI, − 0.94 to − 0.40) for rectal resection, and − 0.33 days (95% CI, − 0.42 to − 0.24) for radical nephrectomy (all *p* < 0.05; Table 4).

**Table 3:** Inverse probability treatment weighting (IPTW)-Adjusted outcomes: Open vs. Minimally invasive surgical approach (MIS)

	LOS, day				Reoperation, %			Discharge to home, %			Readmission, %		
	Median (Q1, Q3)	Mean ± SD	Adj Diff [95% CI]	*P* value	*N* (%)	Adj Ratio [95% CI]	*P* value	*N* (%)	Adj Ratio [95% CI]	*P* value	*N* (%)	Adj Ratio [95% CI]	*P* value
Lobectomy													
Open	6 (4, 8)	7.4 ± 5.3	NA	NA	530 (4.6)	NA	NA	10,073 (87.6)	NA	NA	911 (7.9)	NA	NA
MIS	4 (3, 7)	5.5 ± 4.6	− 1.87 [− 1.99, − 1.75]	< 0.001	707 (3.3)	0.71 [0.63, 0.80]	< 0.001	19,721 (91.7)	1.54 [1.43, 1.65]	< 0.001	1,452 (6.7)	0.84 [0.77, 0.92]	< 0.001
Colon resection													
Open	5 (4, 7)	6.3 ± 5.0	NA	NA	531 (3.0)	NA	NA	15,244 (87.0)	NA	NA	1,598 (9.1)	NA	NA
MIS	4 (3, 6)	4.9 ± 4.1	− 1.34 [− 1.43, − 1.26]	< 0.001	813 (2.4)	0.78 [0.69, 0.87]	< 0.001	31,460 (91.3)	1.58 [1.49, 1.68]	< 0.001	2,436 (7.1)	0.76 [0.71, 0.81]	< 0.001
Rectal resection													
Open	6 (4, 8)	7.0 ± 5.8	NA	NA	171 (4.2)	NA	NA	3,643 (88.5)	NA	NA	626 (15.2)	NA	NA
MIS	5 (3, 7)	6.5 ± 5.9	− 0.47 [− 0.70, − 0.24]	< 0.001	324 (4.7)	1.13 [0.94, 1.37]	0.212	6,219 (89.7)	1.12 [0.99, 1.27]	0.066	1,033 (14.9)	0.98 [0.88, 1.09]	0.643
Radical nephrectomy													
Open	4 (3, 5)	4.7 ± 4.2	NA	NA	142 (1.5)	NA	NA	8,941 (91.5)	NA	NA	554 (5.7)	NA	NA
MIS	3 (2, 4)	3.5 ± 3.1	− 1.21 [− 1.30, − 1.11]	< 0.001	175 (1.1)	0.72 [0.58, 0.90]	0.004	15,605 (93.9)	1.45 [1.32, 1.59]	< 0.001	830 (5.0)	0.88 [0.78, 0.98]	0.019

**Table 4:** Inverse probability treatment weighting (IPTW)-Adjusted outcomes: Laparoscopic/Video-assisted thoracoscopic surgery (LAP/VATS) vs. Robotic (RAS)

	LOS, day				Reoperation, %			Discharge to home, %			Readmission, %		
	Median (Q1, Q3)	Mean ± SD	Adj Diff [95% CI]	*P* value	*N* (%)	Adj Ratio [95% CI]	*P* value	*N* (%)	Adj Ratio [95% CI]	*P* value	*N* (%)	Adj Ratio [95% CI]	*P* value
Lobectomy−													
VATS	4 (3, 6)	5.3 ± 4.4	NA	NA	383 (2.9)	NA	NA	12,263 (92.5)	NA	NA	897 (6.8)	NA	NA
RAS	4 (3, 6)	5.1 ± 4.7	−0.13 [− 0.25, − 0.01]	0.041	258 (3.2)	1.12 [0.95, 1.31]	0.167	7,351 (91.9)	0.93 [0.84, 1.03]	0.15	524 (6.6)	0.97 [0.86, 1.08]	0.548
Colon resection													
LAP	4 (3, 6)	4.9 ± 4.1	NA	NA	579 (2.2)	NA	NA	23,757 (91.9)	NA	NA	1,808 (7.0)	NA	NA
RAS	4 (3, 5)	4.6 ± 4.2	− 0.28 [− 0.37, − 0.18]	< 0.001	210 (2.4)	1.09 [0.93, 1.28]	0.273	7,891 (91.9)	1.00 [0.92, 1.10]	0.980	596 (6.9)	0.99 [0.90, 1.09]	0.867
Rectal resection													
LAP	5 (4, 8)	6.7 ± 5.8	NA	NA	142 (4.4)	NA	NA	2,909 (89.0)	NA	NA	505 (15.5)	NA	NA
RAS	4 (3, 7)	6.0 ± 5.6	− 0.67 [− 0.94, − 0.40]	< 0.001	169 (4.6)	1.06 [0.84, 1.33]	0.649	3,355 (91.2)	1.28 [1.09, 1.50]	0.002	505 (13.7)	0.87 [0.76, 1.00]	0.041
Radical nephrectomy													
LAP	3 (2, 4)	3.6 ± 3.1	NA	NA	86 (1.0)	NA	NA	8,206 (93.7)	NA	NA	431 (4.9)	NA	NA
RAS	3 (2, 4)	3.2 ± 2.9	− 0.33 [− 0.42, − 0.24]	< 0.001	77 (1.0)	1.00 [0.73, 1.36]	1.000	7,367 (94.5)	1.15 [1.01, 1.31]	0.035	369 (4.7)	0.96 [0.83, 1.11]	0.575

Compared to open patients, MIS patients were less likely to have a reoperation (OR for lobectomy: 0.71 [0.63, 0.80], *p* < 0.001; colon resection: 0.78 [0.69, 0.87], *p* < 0.001; radical nephrectomy: 0.72 [0.58, 0.90], *p* = 0.004) and more likely to discharge to home (OR for lobectomy: 1.54 [1.43, 1.65], *p* < 0.001; colon resection: 1.58 [1.49, 1.68], *p* < 0.001; radical nephrectomy: 1.45 [1.32, 1.59], *p* < 0.001) except for rectal resection. Compared to laparoscopic approach, RAS had increased odds of discharge to home in rectal resection (OR: 1.28 [1.09, 1.50], *p* = 0.002) and radical nephrectomy (OR: 1.15 [1.01, 1.31], *p* = 0.035), while no difference in reoperation.

Patients who underwent MIS approach had 12% to 24% lower odds of readmission compared to open surgery during the first 30 days after discharge for lobectomy (OR: 0.84 [0.77, 0.92], *p* < 0.001), colon resection (OR: 0.76 [0.71, 0.81], *p* < 0.001), and radical nephrectomy (OR: 0.88 [0.78, 0.98], *p* = 0.019). Robotic rectal resection reduced the odds of 30-day readmission by 13% (OR: 0.87 [0.76, 1.00], *p* = 0.041) compared to laparoscopic surgery.

In the sensitivity analysis, MIS significantly decreased odds of ICU admission and mechanical ventilation use compared to open surgery in lobectomy, colon resection, and radical nephrectomy (Supplementary Table 4; all *p* < 0.001). MIS rectal resection was associated with a lower odds of ICU admission compared to open surgery but not mechanical ventilation usage. Within MIS, robotic patients were less likely to convert to open surgery than LAP/VATS approach, except for radical nephrectomy.

## Discussion

As the US healthcare system moves towards value-based healthcare, hospitals and surgeons have increased efforts to improve quality of care and reduce unnecessary resource utilization while achieving the goal of the procedure [11, 12]. Hospital LOS is a common indicator for episode resource use, and readmission after surgery is often viewed as a quality measure by Medicare and other insurers. Our data demonstrates a significant outcomes advantage for MIS procedures compared to open procedures in patients with lung, kidney, and colorectal cancer. MIS is associated with shorter LOS, higher discharge to home rate, and lower rates of reoperation and readmission. Patients who underwent robotic procedures had further reductions in LOS compared to laparoscopic approach, while simultaneously not increasing readmission rates. These data demonstrate substantial outcomes gains for patients who undergo robotic procedures across cancer diagnoses.

As previously described, there has been substantial growth in robotic procedures throughout the world. In a review of data from the OptumLabs Data Warehouse in the United States and the Hospital Episodes Statistics in England, investigators demonstrated that robotic surgery has become the standard approach for radical prostatectomy in the United States and England [13]. Similarly, utilization of robotic proctectomy for rectal cancer has also steadily increased [14]. Confirming this practice change, our generalizable data reveal rapid gains in adoption of robotic procedures across cancer types by study end. With this rapid acceptance, we identified substantial advantages in LOS and open surgery conversions for robotic procedures as compared to open or laparoscopic procedures without additional readmission risk for cancers of the colon, rectum, lung, or kidney.

Length of stay advantages are linked to enhanced recovery, lower costs, and patient satisfaction. The current study showed that MIS patients had fewer reoperations, ICU admissions, and mechanical ventilation use during hospitalization along with shorter LOS. Reductions in hospital and ICU stay have been emphasized during the COVID-19 pandemic to better distribute resources and reserve beds for other care needs. However, reductions in LOS for robotic procedures have not been consistently reported in prior analyses. For example, in an analysis of patients with rectal cancer investigators reviewed claims data from 2005 through 2017, reporting decreased LOS for robotic surgery as compared to open surgery [13]. In contrast, although the lung cancer literature reveals reductions in hospital LOS for minimally invasive approaches as compared to open lung surgery [8], analyses of robotic lung surgery have not demonstrated appreciable gains in LOS as compared to VATS [2, 15]. In kidney cancer, reduced LOS has been demonstrated for MIS vs open modalities, however the literature comparing robotic and laparoscopic modalities has demonstrated inconsistent results [16–19]. In contrast to these data, we can confirm a clear and consistent length of stay advantage for cancers of the colon, rectum, lung, and kidney approached in a robotic fashion.

Some of the LOS benefit for robotically approached procedures may be related to fewer conversions from minimally invasive to open surgery. In an analysis of administrative data including patients who underwent right colectomy, investigators found that patients who underwent robotic as compared to laparoscopic surgery were significantly less likely to undergo conversion [4]. Similarly, data from the Norwegian Registry for Gastrointestinal Surgery and from the Norwegian Colorectal Cancer Registry also revealed lower conversion rates with robotic-assisted rectal resections compared with conventional laparoscopic resections [20]. In the same manner, meta-analyses of patients with lung cancer have similarly identified lower conversion to open surgery for patients who underwent robotic surgery as compared to video-assisted surgery [15, 21]. Although not all studies have demonstrated fewer conversions with robotic surgery [7], our data convincingly demonstrate significant reductions in conversions in all studied procedures except for nephrectomy. In fact, for rectal, colon, and lung cancer, our data reveal substantial reductions in conversions across the board. Given that minimally invasive conversions are reportedly associated with higher rates of postoperative complications [20] and increased length of stay, we propose reductions in conversion as a potential mechanism for robotic length of stay improvement.

Another variable that may be contributing to the significant reduction in LOS for MIS and especially robotic procedures relate to less pain and decreased dependency on opioids in post-operative care. Several studies have reported that better pain management reduces hospital length of stay [22, 23]. MIS, especially robotic-assisted surgery, has been observed to have lower post-operative opioids use across multiple clinical specialties. In an analysis of thoracic lobectomy procedures from the Premier database, robotic patients received opioids less frequently, and with lower total and average daily doses, compared to those undergoing VATS and open procedures [24]. In a similar analysis of sigmoidectomies, robotic patients were administered lower doses of parenteral opioids in comparison to open or laparoscopic patients [25]. These findings are consistent with the results of an analysis within our own institution, where we found that minimally invasive techniques were associated with a reduced risk of prolonged opioid use [26].

Our study identified lower readmission rates when patients underwent minimally invasive procedures for colorectal, lung, and kidney cancer, with additional improvements for those patients who underwent robotic procedures. A 2017 study of robotic prostate surgery revealed a decreased LOS and 30-day readmissions for robotic surgery as compared to open surgery [13]. Similarly, reductions in readmission were noted for obese patients with robotic colorectal cancer procedures in a meta-analysis of laparoscopic versus robotic surgery [27]. Historically, shorter length of stay is often linked to higher risk of readmission [28, 29], yet we did not identify an increased risk of readmission in our patients with minimally invasive procedures. Considering the importance of 30-day readmission for payers and policy makers, robotic procedures like other minimally invasive procedures do not seem to lead to a higher risk of readmission.

This study has several limitations. First, this represents a retrospective study of in hospital data without long-term follow-up. However, most acute postoperative complications and deaths often occurred during the initial postoperative period and should largely be captured in these data. Additionally, the policies and protocols regarding postoperative ICU admission may differ significantly across hospital systems with some prophylactically admitting major abdominal or thoracic surgery patients regardless of clinical status. While we could not truly assess hemodynamic status or vasopressor requirement of the patients within this study, the billing code of ICU admission was standardized across all groups and thus may serve as a standard estimate of this variable. Surgeon preference and decision-making for operative approach cannot be completely controlled for and may introduce selection bias in the open surgery though we included several hospital and surgeon characteristics in the IPTW model. Finally, the data and measured outcomes within this study are dependent on appropriate ICD-9-CM, ICD-10, CPT, and billing coding and may be limited by misclassification or data entry error.

In conclusion, our study reveals substantial benefits in robotic surgery for patients with colorectal, lung, and kidney cancer. Many of the outcomes benefits for robotic procedures are shared by patients who undergo minimally invasive procedures, but the additional length of stay benefits are considerable. These additional outcomes benefits are without detriments in readmission, which is of particular importance when understanding downstream treatment effects. It is for these reasons that we can advise that there are both short term and sustained benefits to robotic procedures in the surgical treatment of cancer.

## Supplementary Information

Below is the link to the electronic supplementary material.

Supplementary file1 (DOCX 70 KB)
