# Supplementary material for: Healthcare Resource Utilization After Surgical Treatment of Cancer: Value of Minimally Invasive Surgery
Source: Surg Endosc. 2022 Apr 21;36(10):7549–60. doi: 10.1007/s00464-022-09189-8 (PMC9022614; doi:10.1007/s00464-022-09189-8)
Supplement: Supplementary file 1 — Supplementary file1 (DOCX 70 KB) [file 464_2022_9189_MOESM1_ESM.docx]

**Supplemental Table Content**

[Sup Table 1. Procedure Code list 2](#_Toc81295662)

[Sup Table 2. Demographic and preoperative characteristics, Open vs. MIS: After IPTW 7](#_Toc81295663)

[Sup Table 3. Demographic and preoperative characteristics, Laparoscopic vs. Robotic: After IPTW 10](#_Toc81295664)

[Sup Table 4. Inverse Probability Treatment Weighting (IPTW)- Adjusted Rates of Conversion, ICU and Ventilation Use: Open vs. MIS and VATS/Lap vs. Robotic 13](#_Toc81295665)

# Sup Table 1. Procedure Code list

| **Procedure** | **Code Type** | **Code** | **Description** |
| --- | --- | --- | --- |
| Lobectomy | CPT | 32480 | Removal of lung, other than pneumonectomy; single lobe (lobectomy) |
|  |  | 32482 | Removal of lung, other than pneumonectomy; 2 lobes (bilobectomy) |
|  |  | 32486 | Removal of lung, other than pneumonectomy; with circumferential resection of segment of bronchus followed by broncho-bronchial anastomosis (sleeve lobectomy) |
|  |  | 32663 | Thoracoscopy, surgical; with lobectomy (single lobe) |
|  |  | 32668 | Thoracoscopy, surgical; with diagnostic wedge resection followed by anatomic lung resection (List separately in addition to code for primary procedure) |
|  |  | 32670 | Thoracoscopy, surgical; with removal of two lobes (bilobectomy) |
|  | ICD 9 Procedure | 32.41 | Thoracoscopic lobectomy of lung |
|  |  | 32.49 | Other lobectomy of lung |
|  | ICD 10 Procedure | 0BTC0ZZ | Resection of Right Upper Lung Lobe, Open Approach |
|  |  | 0BTC4ZZ | Resection of Right Upper Lung Lobe, Percutaneous Endoscopic Approach |
|  |  | 0BTD0ZZ | Resection of Right Middle Lung Lobe, Open Approach |
|  |  | 0BTD4ZZ | Resection of Right Middle Lung Lobe, Percutaneous Endoscopic Approach |
|  |  | 0BTF0ZZ | Resection of Right Lower Lung Lobe, Open Approach |
|  |  | 0BTF4ZZ | Resection of Right Lower Lung Lobe, Percutaneous Endoscopic Approach |
|  |  | 0BTG0ZZ | Resection of Left Upper Lung Lobe, Open Approach |
|  |  | 0BTG4ZZ | Resection of Left Upper Lung Lobe, Percutaneous Endoscopic Approach |
|  |  | 0BTJ0ZZ | Resection of Left Lower Lung Lobe, Open Approach |
|  |  | 0BTJ4ZZ | Resection of Left Lower Lung Lobe, Percutaneous Endoscopic Approach |
| Right Colon Resection | ICD 9 Procedure | 17.32 | Laparoscopic cecectomy |
|  |  | 17.33 | Laparoscopic right hemicolectomy |
|  |  | 45.72 | Open and other cecectomy |
|  |  | 45.73 | Open and other right hemicolectomy |
|  | ICD 10 Procedure | 0DTF0ZZ | Resection of Right Large Intestine, Open Approach |
|  |  | 0DTF4ZZ | Resection of Right Large Intestine, Percutaneous Endoscopic Approach |
|  |  | 0DTH0ZZ | Resection of Cecum, Open Approach |
|  |  | 0DTH4ZZ | Resection of Cecum, Percutaneous Endoscopic Approach |
|  |  | 0DTK0ZZ | Resection of Ascending Colon, Open Approach |
|  |  | 0DTK4ZZ | Resection of Ascending Colon, Percutaneous Endoscopic Approach |
| Left Colon Resection | ICD 9 Procedure | 17.35 | Laparoscopic left hemicolectomy |
|  |  | 17.36 | Laparoscopic sigmoidectomy |
|  |  | 45.75 | Open and other left hemicolectomy |
|  |  | 45.76 | Open and other sigmoidectomy |
|  | ICD 10 Procedure | 0DTG0ZZ | Resection of Left Large Intestine, Open Approach |
|  |  | 0DTG4ZZ | Resection of Left Large Intestine, Percutaneous Endoscopic Approach |
|  |  | 0DTM0ZZ | Resection of Descending Colon, Open Approach |
|  |  | 0DTM4ZZ | Resection of Descending Colon, Percutaneous Endoscopic Approach |
|  |  | 0DTN0ZZ | Resection of Sigmoid Colon, Open Approach |
|  |  | 0DTN4ZZ | Resection of Sigmoid Colon, Percutaneous Endoscopic Approach |
| Rectal Resection | CPT | 44145 | Colectomy, partial; with coloproctostomy (low pelvic anastomosis) |
|  |  | 44146 | Colectomy, partial; with coloproctostomy (low pelvic anastomosis), with colostomy |
|  |  | 44207 | Laparoscopy, surgical; colectomy, partial, with anastomosis, with coloproctostomy (low pelvic anastomosis) |
|  |  | 44208 | Laparoscopy, surgical; colectomy, partial, with anastomosis, with coloproctostomy (low pelvic anastomosis) with colostomy |
|  |  | 44211 | Laparoscopy, surgical; colectomy, total, abdominal, with proctectomy, with ileoanal anastomosis, creation of ileal reservoir (S or J), with loop ileostomy, includes rectal mucosectomy, when performed |
|  |  | 44212 | Laparoscopy, surgical; colectomy, total, abdominal, with proctectomy, with ileostomy |
|  |  | 44213 | Laparoscopy, surgical, mobilization (take-down) of splenic flexure performed in conjunction with partial colectomy (List separately in addition to primary procedure) |
|  |  | 45110 | Proctectomy; complete, combined abdominoperineal, with colostomy |
|  |  | 45111 | Proctectomy; partial resection of rectum, transabdominal approach |
|  |  | 45112 | Proctectomy, combined abdominoperineal, pull-through procedure (eg, colo-anal anastomosis) |
|  |  | 45114 | Proctectomy, partial, with anastomosis; abdominal and transsacral approach |
|  |  | 45116 | Proctectomy, partial, with anastomosis; transsacral approach only (Kraske type) |
|  |  | 45119 | Proctectomy, combined abdominoperineal pull-through procedure (eg, colo-anal anastomosis), with creation of colonic reservoir (eg, J-pouch), with diverting enterostomy when performed |
|  |  | 45123 | Proctectomy, partial, without anastomosis, perineal approach |
|  |  | 45395 | Laparoscopy, surgical; proctectomy, complete, combined abdominoperineal, with colostomy |
|  |  | 45397 | Laparoscopy, surgical; proctectomy, combined abdominoperineal pull-through procedure (eg, colo-anal anastomosis), with creation of colonic reservoir (eg, J-pouch), with diverting enterostomy, when performed |
|  | ICD 9 Procedure | 48.40 | Pull-through resection of rectum, not otherwise specified |
|  |  | 48.41 | Soave submucosal resection of rectum |
|  |  | 48.42 | Laparoscopic pull-through resection of rectum |
|  |  | 48.43 | Open pull-through resection of rectum |
|  |  | 48.49 | Other pull-through resection of rectum |
|  |  | 48.50 | Abdominoperineal resection of the rectum, not otherwise specified |
|  |  | 48.51 | Laparoscopic abdominoperineal resection of the rectum |
|  |  | 48.52 | Open abdominoperineal resection of the rectum |
|  |  | 48.59 | Other abdominoperineal resection of the rectum |
|  |  | 48.61 | Transsacral rectosigmoidectomy |
|  |  | 48.64 | Posterior resection of rectum |
|  |  | 48.65 | Duhamel resection of rectum |
|  |  | 48.69 | Other resection of rectum |
|  | ICD 10 Procedure | 0DBP0ZZ | Excision of Rectum, Open Approach |
|  |  | 0DBP4ZZ | Excision of Rectum, Percutaneous Endoscopic Approach |
|  |  | 0DBP7ZZ | Excision of Rectum, Via Natural or Artificial Opening |
|  |  | 0DBP8ZZ | Excision of Rectum, Via Natural or Artificial Opening Endoscopic |
|  |  | 0DTP0ZZ | Resection of Rectum, Open Approach |
|  |  | 0DTP4ZZ | Resection of Rectum, Percutaneous Endoscopic Approach |
|  |  | 0DTP7ZZ | Resection of Rectum, Via Natural or Artificial Opening |
|  |  | 0DTP8ZZ | Resection of Rectum, Via Natural or Artificial Opening Endoscopic |
| Radical Nephrectomy | CPT | 50230 | Nephrectomy, including partial ureterectomy, any open approach including rib resection; radical, with regional lymphadenectomy and/or vena caval thrombectomy |
|  |  | 50545 | Laparoscopy, surgical; radical nephrectomy (includes removal of Gerota's fascia and surrounding fatty tissue, removal of regional lymph nodes, and adrenalectomy) |
|  | ICD 9 Procedure | 55.51 | Nephroureterectomy |
|  |  | 55.52 | Nephrectomy of remaining kidney |
|  |  | 55.54 | Bilateral nephrectomy |
|  | ICD 10 Procedure | 0TT00ZZ | Resection of Right Kidney, Open Approach |
|  |  | 0TT04ZZ | Resection of Right Kidney, Percutaneous Endoscopic Approach |
|  |  | 0TT10ZZ | Resection of Left Kidney, Open Approach |
|  |  | 0TT14ZZ | Resection of Left Kidney, Percutaneous Endoscopic Approach |
|  |  | 0TT20ZZ | Resection of Bilateral Kidneys, Open Approach |
|  |  | 0TT24ZZ | Resection of Bilateral Kidneys, Percutaneous Endoscopic Approach |
|  |  | 0TT60ZZ | Resection of Right Ureter, Open Approach |
|  |  | 0TT64ZZ | Resection of Right Ureter, Percutaneous Endoscopic Approach |
|  |  | 0TT70ZZ | Resection of Left Ureter, Open Approach |
|  |  | 0TT74ZZ | Resection of Left Ureter, Percutaneous Endoscopic Approach |
| Robotic Approach | CPT | S2900 | HCPCS: Surgical techniques requiring use of robotic surgical system (list separately in addition to code for primary procedure) |
|  | ICD 9 Procedure | 17.41 | Open robotic assisted procedure |
|  |  | 17.42 | Laparoscopic robotic assisted procedure |
|  |  | 17.43 | Percutaneous robotic assisted procedure |
|  |  | 17.44 | Endoscopic robotic assisted procedure |
|  |  | 17.45 | Thoracoscopic robotic assisted procedure |
|  |  | 17.49 | Other and unspecified robotic assisted procedure |
|  | ICD 10 Procedure | 8E090CZ | Robotic Assisted Procedure of Head and Neck Region, Open Approach |
|  |  | 8E093CZ | Robotic Assisted Procedure of Head and Neck Region, Percutaneous Approach |
|  |  | 8E094CZ | Robotic Assisted Procedure of Head and Neck Region, Percutaneous Endoscopic Approach |
|  |  | 8E097CZ | Robotic Assisted Procedure of Head and Neck Region, Via Natural or Artificial Opening |
|  |  | 8E098CZ | Robotic Assisted Procedure of Head and Neck Region, Via Natural or Artificial Opening Endoscopic |
|  |  | 8E09XCZ | Robotic Assisted Procedure of Head and Neck Region |
|  |  | 8E0W0CZ | Robotic Assisted Procedure of Trunk Region, Open Approach |
|  |  | 8E0W3CZ | Robotic Assisted Procedure of Trunk Region, Percutaneous Approach |
|  |  | 8E0W4CZ | Robotic Assisted Procedure of Trunk Region, Percutaneous Endoscopic Approach |
|  |  | 8E0W7CZ | Robotic Assisted Procedure of Trunk Region, Via Natural or Artificial Opening |
|  |  | 8E0W8CZ | Robotic Assisted Procedure of Trunk Region, Via Natural or Artificial Opening Endoscopic |
|  |  | 8E0WXCZ | Robotic Assisted Procedure of Trunk Region |
|  |  | 8E0X0CZ | Robotic Assisted Procedure of Upper Extremity, Open Approach |
|  |  | 8E0X3CZ | Robotic Assisted Procedure of Upper Extremity, Percutaneous Approach |
|  |  | 8E0X4CZ | Robotic Assisted Procedure of Upper Extremity, Percutaneous Endoscopic Approach |
|  |  | 8E0XXCZ | Robotic Assisted Procedure of Upper Extremity |
|  |  | 8E0Y0CZ | Robotic Assisted Procedure of Lower Extremity, Open Approach |
|  |  | 8E0Y3CZ | Robotic Assisted Procedure of Lower Extremity, Percutaneous Approach |
|  |  | 8E0Y4CZ | Robotic Assisted Procedure of Lower Extremity, Percutaneous Endoscopic Approach |
|  |  | 8E0YXCZ | Robotic Assisted Procedure of Lower Extremity |
|  | Billing Record Text Search |  | Search Pattern: '%ENDO%WRIST%', '%ENDO%WRST%', '%ENDWRST%', '%ENDOWRST%', '%ENDOWRIST%', '%ROBOT%', '%INTUITIVE%', '%VINCI%' |

# Sup Table 2. Demographic and preoperative characteristics, Open vs. MIS: After IPTW

| **Characteristic** | **Lobectomy** | | |  | **Colon Resection** | | |  | **Rectal resection** | | |  | **Radical Nephrectomy** | | |
| --- | --- | --- | --- | --- | --- | --- | --- | --- | --- | --- | --- | --- | --- | --- | --- |
|  | **Open**, N = 11,504 | **MIS**, N = 21,510 | **Std Diff** |  | **Open**, N = 17,529 | **MIS**, N = 34,443 | **Std Diff** |  | **Open**, N = 4,114 | **MIS**, N = 6,935 | **Std Diff** |  | **Open**, N = 9,776 | **MIS**, N = 16,611 | **Std Diff** |
| **Age groups, n (%)** |  |  |  |  |  |  |  |  |  |  |  |  |  |  |  |
| 18-44 years | 112 (1.0) | 208 (1.0) | 0.001 |  | 715 (4.1) | 1,395 (4.1) | 0.001 |  | 287 (7.0) | 486 (7.0) | 0.002 |  | 638 (6.5) | 1,084 (6.5) | 0.000 |
| 45-54 years | 749 (6.5) | 1,411 (6.6) | 0.002 |  | 2,191 (12.5) | 4,343 (12.6) | 0.003 |  | 830 (20.2) | 1,406 (20.3) | 0.003 |  | 1,478 (15.1) | 2,549 (15.3) | 0.006 |
| 55-64 years | 3,066 (26.7) | 5,657 (26.3) | 0.008 |  | 3,776 (21.5) | 7,419 (21.5) | 0.000 |  | 1,203 (29.2) | 2,021 (29.1) | 0.002 |  | 2,674 (27.4) | 4,532 (27.3) | 0.002 |
| 65+ | 7,572 (65.8) | 14,236 (66.2) | 0.007 |  | 10,847 (61.9) | 21,286 (61.8) | 0.002 |  | 1,796 (43.6) | 3,021 (43.6) | 0.002 |  | 4,987 (51.0) | 8,446 (50.8) | 0.003 |
| **Gender, Male, n (%)** | 5,244 (45.6) | 9,883 (45.9) | 0.007 |  | 8,612 (49.1) | 16,863 (49.0) | 0.003 |  | 2,519 (61.2) | 4,241 (61.2) | 0.001 |  | 6,145 (62.9) | 10,448 (62.9) | 0.001 |
| **Race/ethnicity, n (%)** |  |  |  |  |  |  |  |  |  |  |  |  |  |  |  |
| White | 9,597 (83.5) | 18,008 (83.7) | 0.007 |  | 13,819 (78.8) | 27,103 (78.7) | 0.004 |  | 3,299 (80.2) | 5,556 (80.1) | 0.002 |  | 7,485 (76.6) | 12,721 (76.6) | 0.000 |
| African American | 873 (7.6) | 1,587 (7.4) | 0.008 |  | 1,741 (9.9) | 3,499 (10.2) | 0.008 |  | 292 (7.1) | 495 (7.1) | 0.002 |  | 934 (9.6) | 1,579 (9.5) | 0.002 |
| Hispanic | 466 (4.1) | 775 (3.6) | 0.025 |  | 908 (5.2) | 1,710 (5.0) | 0.010 |  | 253 (6.2) | 424 (6.1) | 0.002 |  | 666 (6.8) | 1,130 (6.8) | 0.001 |
| Other | 563 (4.9) | 1,141 (5.3) | 0.018 |  | 1,062 (6.1) | 2,130 (6.2) | 0.005 |  | 270 (6.6) | 460 (6.6) | 0.003 |  | 690 (7.1) | 1,181 (7.1) | 0.002 |
| **Insurance type, n (%)** |  |  |  |  |  |  |  |  |  |  |  |  |  |  |  |
| Medicare | 7,675 (66.7) | 14,398 (66.9) | 0.004 |  | 10,546 (60.2) | 20,706 (60.1) | 0.001 |  | 1,776 (43.2) | 2,985 (43.0) | 0.002 |  | 5,198 (53.2) | 8,801 (53.0) | 0.004 |
| Medicaid | 761 (6.6) | 1,363 (6.3) | 0.011 |  | 905 (5.2) | 1,786 (5.2) | 0.001 |  | 426 (10.4) | 710 (10.2) | 0.004 |  | 694 (7.1) | 1,171 (7.1) | 0.002 |
| Commercial | 2,654 (23.1) | 5,002 (23.3) | 0.004 |  | 5,448 (31.1) | 10,714 (31.1) | 0.001 |  | 1,707 (41.5) | 2,891 (41.7) | 0.004 |  | 3,429 (35.1) | 5,865 (35.3) | 0.005 |
| Other | 410 (3.6) | 749 (3.5) | 0.005 |  | 630 (3.6) | 1,237 (3.6) | 0.000 |  | 206 (5.0) | 350 (5.0) | 0.002 |  | 455 (4.7) | 773 (4.7) | 0.000 |
| **Charlson Comorbidity Score (No cancer), n (%)** |  |  |  |  |  |  |  |  |  |  |  |  |  |  |  |
| Cci = 0 | 3,862 (33.6) | 7,193 (33.4) | 0.003 |  | 9,404 (53.7) | 18,430 (53.5) | 0.003 |  | 2,485 (60.4) | 4,188 (60.4) | 0.000 |  | 5,455 (55.8) | 9,252 (55.7) | 0.002 |
| Cci = 1 | 4,485 (39.0) | 8,423 (39.2) | 0.003 |  | 2,358 (13.5) | 4,643 (13.5) | 0.001 |  | 496 (12.1) | 838 (12.1) | 0.001 |  | 1,879 (19.2) | 3,188 (19.2) | 0.001 |
| Cci >= 2 | 3,152 (27.4) | 5,896 (27.4) | 0.000 |  | 5,766 (32.9) | 11,370 (33.0) | 0.002 |  | 1,133 (27.5) | 1,909 (27.5) | 0.000 |  | 2,442 (25.0) | 4,171 (25.1) | 0.003 |
| **Metastasis, n (%)** | 1,535 (13.3) | 2,866 (13.3) | 0.001 |  | 3,431 (19.6) | 6,758 (19.6) | 0.001 |  | 765 (18.6) | 1,285 (18.5) | 0.002 |  | 893 (9.1) | 1,521 (9.2) | 0.001 |
| **Obese or overweight, n (%)** | 1,592 (13.8) | 2,985 (13.9) | 0.001 |  | 3,378 (19.3) | 6,663 (19.3) | 0.002 |  | 710 (17.3) | 1,203 (17.4) | 0.002 |  | 2,163 (22.1) | 3,694 (22.2) | 0.003 |
| **Current or former smoker, n (%)** | 8,894 (77.3) | 16,683 (77.6) | 0.005 |  | 6,314 (36.0) | 12,465 (36.2) | 0.004 |  | 1,622 (39.4) | 2,725 (39.3) | 0.003 |  | 3,874 (39.6) | 6,659 (40.1) | 0.009 |
| **Surgeon specialty, n (%)** |  |  |  |  |  |  |  |  |  |  |  |  |  |  |  |
| Procedure specialist | 9,652 (83.9) | 18,154 (84.4) | 0.013 |  | 5,025 (28.7) | 9,784 (28.4) | 0.006 |  | 1,973 (48.0) | 3,337 (48.1) | 0.003 |  | 9,116 (93.2) | 15,498 (93.3) | 0.002 |
| General surgery | 874 (7.6) | 1,657 (7.7) | 0.004 |  | 10,656 (60.8) | 20,998 (61.0) | 0.004 |  | 1,767 (42.9) | 2,964 (42.7) | 0.004 |  | 79 (0.8) | 129 (0.8) | 0.003 |
| Other/Unknown | 973 (8.5) | 1,700 (7.9) | 0.021 |  | 1,848 (10.5) | 3,662 (10.6) | 0.003 |  | 374 (9.1) | 634 (9.1) | 0.001 |  | 582 (5.9) | 984 (5.9) | 0.001 |
| **Surgeon volume, n (%)** |  |  |  |  |  |  |  |  |  |  |  |  |  |  |  |
| Low | 4,252 (37.0) | 7,204 (33.5) | 0.075 |  | 5,013 (28.6) | 9,840 (28.6) | 0.001 |  | 983 (23.9) | 1,648 (23.8) | 0.003 |  | 3,579 (36.6) | 5,962 (35.9) | 0.015 |
| Medium | 3,896 (33.9) | 6,998 (32.5) | 0.029 |  | 5,951 (33.9) | 11,693 (33.9) | 0.000 |  | 1,240 (30.1) | 2,088 (30.1) | 0.001 |  | 3,844 (39.3) | 6,455 (38.9) | 0.009 |
| High | 3,351 (29.1) | 7,310 (34.0) | 0.111 |  | 6,565 (37.5) | 12,911 (37.5) | 0.001 |  | 1,891 (46.0) | 3,199 (46.1) | 0.003 |  | 2,353 (24.1) | 4,194 (25.3) | 0.028 |
| **Hospital volume, n (%)** |  |  |  |  |  |  |  |  |  |  |  |  |  |  |  |
| Low | 4,133 (35.9) | 7,300 (33.9) | 0.043 |  | 6,191 (35.3) | 12,283 (35.7) | 0.007 |  | 962 (23.4) | 1,628 (23.5) | 0.002 |  | 4,063 (41.6) | 6,812 (41.0) | 0.011 |
| Medium | 4,411 (38.4) | 7,473 (34.7) | 0.076 |  | 6,072 (34.6) | 11,942 (34.7) | 0.001 |  | 1,477 (35.9) | 2,490 (35.9) | 0.000 |  | 3,304 (33.8) | 5,542 (33.4) | 0.009 |
| High | 2,956 (25.7) | 6,739 (31.3) | 0.129 |  | 5,266 (30.0) | 10,217 (29.7) | 0.008 |  | 1,675 (40.7) | 2,818 (40.6) | 0.002 |  | 2,409 (24.6) | 4,257 (25.6) | 0.023 |
| **Teaching hospital, n (%)** | 6,302 (54.8) | 12,432 (57.8) | 0.061 |  | 8,195 (46.8) | 16,005 (46.5) | 0.006 |  | 2,232 (54.2) | 3,747 (54.0) | 0.004 |  | 4,977 (50.9) | 8,534 (51.4) | 0.009 |
| **Urban Region, n (%)** | 10,693 (93.0) | 19,841 (92.2) | 0.028 |  | 15,385 (87.8) | 30,171 (87.6) | 0.005 |  | 3,742 (90.9) | 6,300 (90.8) | 0.004 |  | 8,924 (91.3) | 15,146 (91.2) | 0.004 |
| **Geographic region, n (%)** |  |  |  |  |  |  |  |  |  |  |  |  |  |  |  |
| Midwest | 2,504 (21.8) | 4,893 (22.7) | 0.023 |  | 4,056 (23.1) | 7,914 (23.0) | 0.004 |  | 951 (23.1) | 1,609 (23.2) | 0.002 |  | 2,122 (21.7) | 3,685 (22.2) | 0.012 |
| Northeast | 1,989 (17.3) | 3,554 (16.5) | 0.022 |  | 2,813 (16.0) | 5,492 (15.9) | 0.003 |  | 596 (14.5) | 994 (14.3) | 0.004 |  | 1,479 (15.1) | 2,466 (14.8) | 0.008 |
| South | 5,366 (46.7) | 10,051 (46.7) | 0.001 |  | 8,055 (46.0) | 15,944 (46.3) | 0.007 |  | 1,875 (45.6) | 3,172 (45.7) | 0.003 |  | 4,580 (46.8) | 7,789 (46.9) | 0.001 |
| West | 1,640 (14.3) | 3,013 (14.0) | 0.007 |  | 2,605 (14.9) | 5,093 (14.8) | 0.002 |  | 692 (16.8) | 1,161 (16.7) | 0.002 |  | 1,595 (16.3) | 2,670 (16.1) | 0.007 |
| **Hospital bed size, n (%)** |  |  |  |  |  |  |  |  |  |  |  |  |  |  |  |
| 0-299 beds | 2,116 (18.4) | 3,908 (18.2) | 0.006 |  | 5,828 (33.3) | 11,494 (33.4) | 0.003 |  | 1,032 (25.1) | 1,745 (25.2) | 0.002 |  | 2,726 (27.9) | 4,588 (27.6) | 0.006 |
| 300-499 beds | 4,019 (35.0) | 7,004 (32.6) | 0.051 |  | 5,230 (29.8) | 10,332 (30.0) | 0.004 |  | 1,277 (31.0) | 2,157 (31.1) | 0.002 |  | 2,958 (30.3) | 5,037 (30.3) | 0.001 |
| 500+ beds | 5,364 (46.6) | 10,599 (49.3) | 0.053 |  | 6,470 (36.9) | 12,616 (36.6) | 0.006 |  | 1,805 (43.9) | 3,033 (43.7) | 0.003 |  | 4,092 (41.9) | 6,986 (42.1) | 0.004 |
| **Year of surgery, n (%)** |  |  |  |  |  |  |  |  |  |  |  |  |  |  |  |
| 2014 | 1,941 (16.9) | 3,523 (16.4) | 0.013 |  | 3,176 (18.1) | 6,275 (18.2) | 0.003 |  | 610 (14.8) | 1,020 (14.7) | 0.004 |  | 1,747 (17.9) | 2,920 (17.6) | 0.008 |
| 2015 | 2,057 (17.9) | 3,747 (17.4) | 0.012 |  | 3,051 (17.4) | 6,081 (17.7) | 0.007 |  | 693 (16.8) | 1,163 (16.8) | 0.002 |  | 1,812 (18.5) | 2,995 (18.0) | 0.013 |
| 2016 | 2,058 (17.9) | 3,658 (17.0) | 0.024 |  | 2,915 (16.6) | 5,757 (16.7) | 0.002 |  | 822 (20.0) | 1,382 (19.9) | 0.001 |  | 1,594 (16.3) | 2,732 (16.4) | 0.004 |
| 2017 | 1,838 (16.0) | 3,652 (17.0) | 0.026 |  | 2,992 (17.1) | 5,858 (17.0) | 0.002 |  | 744 (18.1) | 1,259 (18.1) | 0.002 |  | 1,606 (16.4) | 2,797 (16.8) | 0.011 |
| 2018 | 1,912 (16.6) | 3,611 (16.8) | 0.004 |  | 2,769 (15.8) | 5,423 (15.7) | 0.001 |  | 661 (16.1) | 1,128 (16.3) | 0.006 |  | 1,528 (15.6) | 2,605 (15.7) | 0.001 |
| 2019 | 1,693 (14.7) | 3,320 (15.4) | 0.020 |  | 2,626 (15.0) | 5,049 (14.7) | 0.009 |  | 584 (14.2) | 984 (14.2) | 0.001 |  | 1,488 (15.2) | 2,563 (15.4) | 0.006 |
| **Colon resection type, n (%)** |  |  |  |  |  |  |  |  |  |  |  |  |  |  |  |
| Left colectomy | NA | NA |  |  | 5,996 (34.2) | 11,758 (34.1) | 0.001 |  | NA | NA |  |  | NA | NA |  |
| Right colectomy | NA | NA |  |  | 11,533 (65.8) | 22,685 (65.9) | 0.001 |  | NA | NA |  |  | NA | NA |  |

# Sup Table 3. Demographic and preoperative characteristics, Laparoscopic vs. Robotic: After IPTW

| **Characteristic** | **Lobectomy** | | | |  | | **Colon Resection** | | | |  | | **Rectal resection** | | | |  | | **Radical Nephrectomy** | | |
| --- | --- | --- | --- | --- | --- | --- | --- | --- | --- | --- | --- | --- | --- | --- | --- | --- | --- | --- | --- | --- | --- |
|  | **Laparoscopic**, N = 13,259 | **Robotic**, N = 7,995 | **Std Diff** |  | | **Laparoscopic**, N = 25,864 | | **Robotic**, N = 8,590 | **Std Diff** |  | | **Laparoscopic**, N = 3,270 | | **Robotic**, N = 3,680 | **Std Diff** |  | | **Laparoscopic**, N = 8,758 | | **Robotic**, N = 7,798 | **Std Diff** |
| **Age groups, n (%)** |  |  |  |  | |  | |  |  |  | |  | |  |  |  | |  | |  |  |
| 18-44 years | 119 (0.9) | 73 (0.9) | 0.001 |  | | 1,065 (4.1) | | 355 (4.1) | 0.001 |  | | 235 (7.2) | | 269 (7.3) | 0.004 |  | | 593 (6.8) | | 523 (6.7) | 0.003 |
| 45-54 years | 803 (6.1) | 476 (6.0) | 0.004 |  | | 3,484 (13.5) | | 1,171 (13.6) | 0.005 |  | | 703 (21.5) | | 791 (21.5) | 0.001 |  | | 1,327 (15.2) | | 1,202 (15.4) | 0.007 |
| 55-64 years | 3,359 (25.3) | 2,044 (25.6) | 0.005 |  | | 5,678 (22.0) | | 1,910 (22.2) | 0.007 |  | | 957 (29.3) | | 1,068 (29.0) | 0.006 |  | | 2,368 (27.0) | | 2,112 (27.1) | 0.001 |
| 65+ | 8,978 (67.7) | 5,402 (67.6) | 0.003 |  | | 15,637 (60.5) | | 5,154 (60.0) | 0.009 |  | | 1,374 (42.0) | | 1,553 (42.2) | 0.004 |  | | 4,469 (51.0) | | 3,961 (50.8) | 0.005 |
| **Gender, Male, n (%)** | 5,848 (44.1) | 3,523 (44.1) | 0.001 |  | | 12,779 (49.4) | | 4,254 (49.5) | 0.002 |  | | 2,008 (61.4) | | 2,262 (61.5) | 0.001 |  | | 5,476 (62.5) | | 4,886 (62.7) | 0.003 |
| **Race/ethnicity, n (%)** |  |  |  |  | |  | |  |  |  | |  | |  |  |  | |  | |  |  |
| White | 11,026 (83.2) | 6,643 (83.1) | 0.002 |  | | 20,235 (78.2) | | 6,703 (78.0) | 0.005 |  | | 2,614 (80.0) | | 2,935 (79.7) | 0.005 |  | | 6,672 (76.2) | | 5,911 (75.8) | 0.009 |
| African American | 1,002 (7.6) | 598 (7.5) | 0.003 |  | | 2,615 (10.1) | | 869 (10.1) | 0.000 |  | | 235 (7.2) | | 265 (7.2) | 0.000 |  | | 840 (9.6) | | 755 (9.7) | 0.003 |
| Hispanic | 575 (4.3) | 346 (4.3) | 0.001 |  | | 1,383 (5.3) | | 483 (5.6) | 0.012 |  | | 191 (5.9) | | 213 (5.8) | 0.003 |  | | 623 (7.1) | | 570 (7.3) | 0.008 |
| Other | 656 (4.9) | 408 (5.1) | 0.007 |  | | 1,631 (6.3) | | 535 (6.2) | 0.003 |  | | 229 (7.0) | | 267 (7.3) | 0.011 |  | | 623 (7.1) | | 562 (7.2) | 0.003 |
| **Insurance type, n (%)** |  |  |  |  | |  | |  |  |  | |  | |  |  |  | |  | |  |  |
| Medicare | 8,972 (67.7) | 5,407 (67.6) | 0.001 |  | | 15,168 (58.6) | | 5,004 (58.3) | 0.008 |  | | 1,347 (41.2) | | 1,524 (41.4) | 0.004 |  | | 4,647 (53.1) | | 4,126 (52.9) | 0.003 |
| Medicaid | 765 (5.8) | 455 (5.7) | 0.003 |  | | 1,234 (4.8) | | 417 (4.9) | 0.004 |  | | 320 (9.8) | | 359 (9.8) | 0.001 |  | | 603 (6.9) | | 544 (7.0) | 0.004 |
| Commercial | 3,109 (23.5) | 1,896 (23.7) | 0.006 |  | | 8,568 (33.1) | | 2,862 (33.3) | 0.004 |  | | 1,439 (44.0) | | 1,613 (43.8) | 0.004 |  | | 3,119 (35.6) | | 2,785 (35.7) | 0.002 |
| Other | 413 (3.1) | 238 (3.0) | 0.008 |  | | 894 (3.5) | | 306 (3.6) | 0.006 |  | | 163 (5.0) | | 184 (5.0) | 0.000 |  | | 389 (4.4) | | 343 (4.4) | 0.002 |
| **Charlson Comorbidity Score (No cancer), n (%)** |  |  |  |  | |  | |  |  |  | |  | |  |  |  | |  | |  |  |
| Cci = 0 | 4,797 (36.2) | 2,897 (36.2) | 0.001 |  | | 14,666 (56.7) | | 4,882 (56.8) | 0.003 |  | | 2,030 (62.1) | | 2,287 (62.1) | 0.001 |  | | 5,022 (57.3) | | 4,472 (57.3) | 0.000 |
| Cci = 1 | 5,101 (38.5) | 3,062 (38.3) | 0.004 |  | | 3,527 (13.6) | | 1,168 (13.6) | 0.001 |  | | 386 (11.8) | | 434 (11.8) | 0.001 |  | | 1,731 (19.8) | | 1,532 (19.6) | 0.003 |
| Cci >= 2 | 3,361 (25.3) | 2,036 (25.5) | 0.003 |  | | 7,671 (29.7) | | 2,540 (29.6) | 0.002 |  | | 853 (26.1) | | 960 (26.1) | 0.000 |  | | 2,005 (22.9) | | 1,794 (23.0) | 0.003 |
| **Metastasis, n (%)** | 1,535 (11.6) | 932 (11.7) | 0.003 |  | | 4,297 (16.6) | | 1,437 (16.7) | 0.003 |  | | 562 (17.2) | | 628 (17.1) | 0.003 |  | | 534 (6.1) | | 489 (6.3) | 0.007 |
| **Obese or overweight, n (%)** | 1,780 (13.4) | 1,066 (13.3) | 0.003 |  | | 5,026 (19.4) | | 1,690 (19.7) | 0.006 |  | | 571 (17.5) | | 642 (17.4) | 0.001 |  | | 1,991 (22.7) | | 1,759 (22.6) | 0.004 |
| **Current or former smoker, n (%)** | 10,117 (76.3) | 6,085 (76.1) | 0.005 |  | | 9,264 (35.8) | | 3,106 (36.2) | 0.007 |  | | 1,275 (39.0) | | 1,445 (39.3) | 0.005 |  | | 3,541 (40.4) | | 3,150 (40.4) | 0.001 |
| **Surgeon specialty, n (%)** |  |  |  |  | |  | |  |  |  | |  | |  |  |  | |  | |  |  |
| Procedure specialist | 11,326 (85.4) | 6,858 (85.8) | 0.010 |  | | 8,354 (32.3) | | 2,820 (32.8) | 0.011 |  | | 1,659 (50.7) | | 1,866 (50.7) | 0.000 |  | | 8,193 (93.6) | | 7,283 (93.4) | 0.007 |
| General surgery | 1,137 (8.6) | 701 (8.8) | 0.007 |  | | 14,931 (57.7) | | 4,864 (56.6) | 0.022 |  | | 1,314 (40.2) | | 1,474 (40.1) | 0.003 |  | | 54 (0.6) | | 43 (0.6) | 0.008 |
| Other/Unknown | 796 (6.0) | 436 (5.5) | 0.024 |  | | 2,579 (10.0) | | 906 (10.6) | 0.020 |  | | 297 (9.1) | | 339 (9.2) | 0.005 |  | | 511 (5.8) | | 472 (6.0) | 0.010 |
| **Surgeon volume, n (%)** |  |  |  |  | |  | |  |  |  | |  | |  |  |  | |  | |  |  |
| Low | 3,355 (25.3) | 2,073 (25.9) | 0.015 |  | | 7,171 (27.7) | | 2,525 (29.4) | 0.037 |  | | 798 (24.4) | | 889 (24.2) | 0.006 |  | | 2,771 (31.6) | | 2,493 (32.0) | 0.008 |
| Medium | 3,944 (29.7) | 2,317 (29.0) | 0.017 |  | | 8,121 (31.4) | | 2,590 (30.2) | 0.027 |  | | 909 (27.8) | | 1,018 (27.7) | 0.003 |  | | 3,352 (38.3) | | 2,933 (37.6) | 0.014 |
| High | 5,960 (44.9) | 3,605 (45.1) | 0.003 |  | | 10,572 (40.9) | | 3,475 (40.5) | 0.009 |  | | 1,562 (47.8) | | 1,772 (48.2) | 0.008 |  | | 2,635 (30.1) | | 2,372 (30.4) | 0.008 |
| **Hospital volume, n (%)** |  |  |  |  | |  | |  |  |  | |  | |  |  |  | |  | |  |  |
| Low | 3,592 (27.1) | 2,190 (27.4) | 0.007 |  | | 8,287 (32.0) | | 2,672 (31.1) | 0.020 |  | | 716 (21.9) | | 839 (22.8) | 0.022 |  | | 3,395 (38.8) | | 3,023 (38.8) | 0.000 |
| Medium | 4,278 (32.3) | 2,551 (31.9) | 0.008 |  | | 9,432 (36.5) | | 3,138 (36.5) | 0.001 |  | | 1,197 (36.6) | | 1,323 (36.0) | 0.013 |  | | 2,852 (32.6) | | 2,526 (32.4) | 0.004 |
| High | 5,388 (40.6) | 3,254 (40.7) | 0.001 |  | | 8,145 (31.5) | | 2,780 (32.4) | 0.019 |  | | 1,357 (41.5) | | 1,518 (41.2) | 0.006 |  | | 2,511 (28.7) | | 2,249 (28.8) | 0.004 |
| **Teaching hospital, n (%)** | 8,382 (63.2) | 5,053 (63.2) | 0.000 |  | | 12,205 (47.2) | | 4,104 (47.8) | 0.012 |  | | 1,764 (54.0) | | 1,960 (53.2) | 0.014 |  | | 4,618 (52.7) | | 4,083 (52.4) | 0.007 |
| **Urban Region, n (%)** | 12,199 (92.0) | 7,368 (92.2) | 0.006 |  | | 23,020 (89.0) | | 7,639 (88.9) | 0.003 |  | | 3,003 (91.8) | | 3,375 (91.7) | 0.005 |  | | 7,959 (90.9) | | 7,099 (91.0) | 0.006 |
| **Geographic region, n (%)** |  |  |  |  | |  | |  |  |  | |  | |  |  |  | |  | |  |  |
| Midwest | 2,781 (21.0) | 1,699 (21.2) | 0.007 |  | | 5,627 (21.8) | | 1,813 (21.1) | 0.016 |  | | 727 (22.2) | | 815 (22.2) | 0.002 |  | | 1,969 (22.5) | | 1,757 (22.5) | 0.001 |
| Northeast | 2,838 (21.4) | 1,718 (21.5) | 0.002 |  | | 4,336 (16.8) | | 1,573 (18.3) | 0.042 |  | | 481 (14.7) | | 542 (14.7) | 0.000 |  | | 1,455 (16.6) | | 1,287 (16.5) | 0.003 |
| South | 5,790 (43.7) | 3,473 (43.4) | 0.005 |  | | 12,018 (46.5) | | 3,931 (45.8) | 0.014 |  | | 1,533 (46.9) | | 1,700 (46.2) | 0.014 |  | | 3,966 (45.3) | | 3,556 (45.6) | 0.006 |
| West | 1,850 (14.0) | 1,105 (13.8) | 0.004 |  | | 3,882 (15.0) | | 1,272 (14.8) | 0.006 |  | | 530 (16.2) | | 623 (16.9) | 0.020 |  | | 1,368 (15.6) | | 1,198 (15.4) | 0.007 |
| **Hospital bed size, n (%)** |  |  |  |  | |  | |  |  |  | |  | |  |  |  | |  | |  |  |
| 0-299 beds | 2,145 (16.2) | 1,260 (15.8) | 0.011 |  | | 8,329 (32.2) | | 2,721 (31.7) | 0.011 |  | | 812 (24.8) | | 933 (25.4) | 0.012 |  | | 2,402 (27.4) | | 2,141 (27.5) | 0.001 |
| 300-499 beds | 3,877 (29.2) | 2,362 (29.5) | 0.007 |  | | 7,604 (29.4) | | 2,531 (29.5) | 0.001 |  | | 1,005 (30.7) | | 1,147 (31.2) | 0.010 |  | | 2,566 (29.3) | | 2,328 (29.9) | 0.012 |
| 500+ beds | 7,237 (54.6) | 4,373 (54.7) | 0.002 |  | | 9,931 (38.4) | | 3,338 (38.9) | 0.009 |  | | 1,453 (44.4) | | 1,600 (43.5) | 0.020 |  | | 3,790 (43.3) | | 3,329 (42.7) | 0.012 |
| **Year of surgery, n (%)** |  |  |  |  | |  | |  |  |  | |  | |  |  |  | |  | |  |  |
| 2014 | 1,881 (14.2) | 1,096 (13.7) | 0.014 |  | | 4,639 (17.9) | | 1,554 (18.1) | 0.005 |  | | 471 (14.4) | | 533 (14.5) | 0.002 |  | | 1,174 (13.4) | | 1,038 (13.3) | 0.003 |
| 2015 | 2,001 (15.1) | 1,194 (14.9) | 0.005 |  | | 4,550 (17.6) | | 1,506 (17.5) | 0.002 |  | | 497 (15.2) | | 565 (15.4) | 0.004 |  | | 1,300 (14.8) | | 1,181 (15.1) | 0.008 |
| 2016 | 2,099 (15.8) | 1,286 (16.1) | 0.007 |  | | 4,053 (15.7) | | 1,337 (15.6) | 0.003 |  | | 596 (18.2) | | 677 (18.4) | 0.005 |  | | 1,552 (17.7) | | 1,394 (17.9) | 0.004 |
| 2017 | 2,341 (17.7) | 1,422 (17.8) | 0.004 |  | | 4,458 (17.2) | | 1,479 (17.2) | 0.001 |  | | 602 (18.4) | | 677 (18.4) | 0.000 |  | | 1,586 (18.1) | | 1,428 (18.3) | 0.005 |
| 2018 | 2,441 (18.4) | 1,485 (18.6) | 0.004 |  | | 4,159 (16.1) | | 1,375 (16.0) | 0.002 |  | | 601 (18.4) | | 664 (18.0) | 0.010 |  | | 1,529 (17.5) | | 1,357 (17.4) | 0.002 |
| 2019 | 2,495 (18.8) | 1,511 (18.9) | 0.002 |  | | 4,005 (15.5) | | 1,339 (15.6) | 0.003 |  | | 503 (15.4) | | 565 (15.4) | 0.001 |  | | 1,617 (18.5) | | 1,400 (18.0) | 0.013 |
| **Colon resection type, n (%)** |  |  |  |  | |  | |  |  |  | |  | |  |  |  | |  | |  |  |
| Left colectomy | NA | NA |  |  | | 8,907 (34.4) | | 2,966 (34.5) | 0.002 |  | | NA | | NA |  |  | | NA | | NA |  |
| Right colectomy | NA | NA |  |  | | 16,956 (65.6) | | 5,623 (65.5) | 0.002 |  | | NA | | NA |  |  | | NA | | NA |  |

# Sup Table 4. Inverse Probability Treatment Weighting (IPTW)- Adjusted Rates of Conversion, ICU and Ventilation Use: Open vs. MIS and VATS/Lap vs. Robotic

|  | **Open vs. MIS** | | | |  | **Lap/VATS vs. RAS** | | | |
| --- | --- | --- | --- | --- | --- | --- | --- | --- | --- |
|  | Open, N (%) | MIS, N (%) | OR [95%] | P-value |  | VATS/Lap, N (%) | RAS, N (%) | OR (95%) | P-value |
| Lobectomy |  |  |  |  |  |  |  |  |  |
| Conversion | NA |  |  |  |  | 1,273 (9.6) | 383 (4.8) | 0.47 [0.42, 0.53] | <0.001 |
| ICU, n (%) | 7,757 (67.5) | 10,215 (47.5) | 0.44 [0.42, 0.46] | <0.001 |  | 5,718 (43.1) | 3,303 (41.3) | 0.93 [0.88, 0.98] | 0.009 |
| ICU≥2d, n (%) | 4,485 (39.0) | 5,307 (24.7) | 0.52 [0.49, 0.54] | <0.001 |  | 3,046 (23.0) | 1,610 (20.1) | 0.85 [0.79, 0.91] | <0.001 |
| Ventilation Use | 730 (6.3) | 886 (4.1) | 0.64 [0.58, 0.71] | <0.001 |  | 503 (3.8) | 293 (3.7%) | 0.96 [0.83, 1.12] | 0.627 |
| Colon resection |  |  |  |  |  |  |  |  |  |
| Conversion | NA |  |  |  |  | 3,012 (11.6) | 535 (6.2) | 0.50 [0.46, 0.55] | <0.001 |
| ICU, n (%) | 2,949 (16.8) | 3,226 (9.4) | 0.51 [0.48, 0.54] | <0.001 |  | 2,205 (8.5) | 857 (10.0) | 1.19 [1.09, 1.29] | <0.001 |
| ICU≥2d, n (%) | 1,920 (11.0) | 2,068 (6.0) | 0.52 [0.49, 0.55] | <0.001 |  | 1,418 (5.5) | 549 (6.4) | 1.18 [1.06, 1.30] | 0.002 |
| Ventilation Use | 525 (3.0) | 744 (2.2) | 0.71 [0.64, 0.80] | <0.001 |  | 520 (2.0) | 194 (2.3) | 1.13 [0.95, 1.33] | 0.163 |
| Rectal resection |  |  |  |  |  |  |  |  |  |
| Conversion | NA |  |  |  |  | 406 (12.4) | 234 (6.4) | 0.48 [0.41, 0.57] | <0.001 |
| ICU, n (%) | 708 (17.2) | 878 (12.7) | 0.70 [0.63, 0.78] | <0.001 |  | 419 (12.8) | 450 (12.2) | 0.95 [0.82, 1.09] | 0.452 |
| ICU≥2d, n (%) | 470 (11.4) | 593 (8.5) | 0.73 [0.64, 0.82] | <0.001 |  | 299 (9.1) | 296 (8.0) | 0.87 [0.74, 1.03] | 0.103 |
| Ventilation Use | 98 (2.4) | 151 (2.2) | 0.91 [0.71, 1.19] | 0.495 |  | 63 (1.9) | 94 (2.6) | 1.34 [0.98, 1.86] | 0.074 |
| Radical nephrectomy |  |  |  |  |  |  |  |  |  |
| Conversion | NA |  |  |  |  | 346 (4.0) | 275 (3.5) | 0.89 [0.75, 1.04] | 0.144 |
| ICU, n (%) | 1,718 (17.6) | 1,447 (8.7) | 0.45 [0.42, 0.48] | <0.001 |  | 779 (8.9) | 645 (8.3) | 0.92 [0.83, 1.03] | 0.153 |
| ICU≥2d, n (%) | 1,072 (11.0) | 820 (4.9) | 0.42 [0.38, 0.46] | <0.001 |  | 423 (4.8) | 363 (4.7) | 0.96 [0.83, 1.11] | 0.604 |
| Ventilation Use | 287 (2.9) | 371 (2.2) | 0.76 [0.65, 0.88] | <0.001 |  | 202 (2.3) | 170 (2.2) | 0.94 [0.77, 1.16] | 0.575 |
